# Lessons learned from COVID-19: improving breast cancer care post-pandemic from the patient perspective

**DOI:** 10.1007/s00520-024-08540-0

**Published:** 2024-05-10

**Authors:** Charlotte Myers, Kathleen Bennett, Caitriona Cahir

**Affiliations:** 1 School of Population Health, RCSI University of Medicine and Health Sciences, 123 St Stephen’s, Green, Dublin, D02 YN77 Ireland; 2 Data Science Centre, School of Population Health, RCSI University of Medicine and Health Sciences, Dublin, Ireland

**Keywords:** COVID-19, Breast cancer, Social determinants of health, Quality of life, Patient voice, Health policy

## Abstract

**Background:**

Since the onset of the pandemic, breast cancer (BC) services have been disrupted in most countries. The purpose of this qualitative study is to explore the unmet needs, patient-priorities, and recommendations for improving BC healthcare post-pandemic for women with BC and to understand how they may vary based on social determinants of health (SDH), in particular socio-economic status (SES).

**Methods:**

Thirty-seven women, who were purposively sampled based on SDH and previously interviewed about the impact of COVID-19 on BC, were invited to take part in follow-up semi-structured qualitative interviews in early 2023. The interviews explored their perspectives of BC care since the easing of COVID-19 government restrictions, including unmet needs, patient-priorities, and recommendations specific to BC care. Thematic analysis was conducted to synthesize each topic narratively with corresponding sub-themes. Additionally, variation by SDH was analyzed within each sub-theme.

**Results:**

Twenty-eight women (mean age = 61.7 years, standard deviation (SD) = 12.3) participated in interviews (response rate = 76%). Thirty-nine percent (*n* = 11) of women were categorized as high-SES, while 61% (*n* = 17) of women were categorized as low-SES. Women expressed unmet needs in their BC care including routine care and mental and physical well-being care, as well as a lack of financial support to access BC care. Patient priorities included the following: developing cohesion between different aspects of BC care; communication with and between healthcare professionals; and patient empowerment within BC care. Recommendations moving forward post-pandemic included improving the transition from active to post-treatment, enhancing support resources, and implementing telemedicine where appropriate. Overall, women of low-SES experienced more severe unmet needs, which in turn resulted in varied patient priorities and recommendations.

**Conclusion:**

As health systems are recovering from the COVID-19 pandemic, the emphasis should be on restoring access to BC care and improving the quality of BC care, with a particular consideration given to those women from low-SES, to reduce health inequalities post-pandemic.

**Supplementary Information:**

The online version contains supplementary material available at 10.1007/s00520-024-08540-0.

## Background

Globally, health services for breast cancer (BC) across the cancer continuum were significantly disrupted and compromised due to the coronavirus disease (COVID-19) pandemic [1]. Breast screening services were curtailed or paused during periods of COVID-19 restrictions [2]. Active cancer treatment, such as surgery, radiotherapy, and chemotherapy, was disrupted and/ or modified to account for the level of restrictions in place [3, 4]. Post-treatment care (i.e., routine care), which includes mammograms, follow-up appointments, blood tests, and other scans, was significantly disrupted during the pandemic [5]. Support services, which address the multi-disciplinary needs of those living with a diagnosis of cancer, including physiotherapy and psycho-oncology, were paused or modified during the pandemic [6, 7].

Breast screening services generally resumed after government restrictions were lifted [8]; however, the impact of pausing breast screening services on BC diagnoses is only now becoming apparent with later stage and more symptomatic BC diagnoses [9] which may have a negative impact on survival rates in the future [10]. Furthermore, the issue of backlogs and waiting lists across the cancer continuum is continuing [11]. There are likely to be considerable unmet needs in healthcare for women with BC due to barriers such as availability, affordability, accessibility, and acceptability of BC services [12] which were apparent during the pandemic. Unmet needs, which can be measured by the difference between required healthcare services and received healthcare services, can assess the effectiveness in healthcare delivery [13]. A failure to address unmet needs can have a negative impact on an individual’s quality of life and other health outcomes [14]. Previous research conducted during the pandemic identified unmet needs for individuals living with a diagnosis of BC such as psychological and emotional support, management of side effects, complementary therapy, communication among healthcare providers, local health care services, and transportation [15]. However, similar research has not been conducted qualitatively post-pandemic to identify lasting unmet needs.

Considering the long-lasting consequences of the pandemic, the acquired knowledge and experience from COVID-19 can be used by healthcare providers and policy makers to improve BC care and to prepare health systems for future unexpected events [16]. Historically, pandemics and other crises have provided opportunities to strengthen health systems by exploiting faults in the pre-existing health system and exacerbating pre-existing health inequalities [17]. For example, low socio-economic status (SES) has been associated with higher disease burden [18] and decreased access to healthcare [19]. Within the context of non-communicable diseases, including BC, the social determinants of health (SDH) framework have been applied to the COVID-19 pandemic to explain health disparities [20]. Specific to BC care, SDH identified during the pandemic include age, race, insurance status, and region (1); however, it is unknown whether these SDH persist post-pandemic. Therefore, health systems should respond to the COVID-19 pandemic by addressing the multidisciplinary and personalized needs of all individuals to improve health equality.

The reorganization of BC services during the pandemic provides an opportunity to improve overall BC care and the experience and priorities of women with BC. To effectively address the needs of individuals, the patient experience and voice is integral for healthcare improvement. The aims of this qualitative study are (i) to explore and identify the unmet needs, patient-priorities, and recommendations for improvements in BC healthcare post-pandemic and (ii) to understand how they may vary for women with BC according to SDH, in particular SES.

## Methods

### Study design

A qualitative exploratory study was conducted using the consolidated criteria for reporting qualitative research (COREQ) guidelines [21].

### Participants

Women with BC were initially enrolled into a prospective cohort study measuring the impact of the COVID-19 pandemic on BC healthcare services and women’s well-being using a questionnaire (*N* = 387) [22]. In total, 37 women from this cohort, purposively sampled based on SDH, were interviewed about their experience of the COVID-19 pandemic on health care and well-being during the pandemic [23]. These 37 women were invited to take part in a follow-up qualitative study to explore the impact of COVID-19 post-pandemic on BC care. The initial SDH sampling strata in the baseline interviews were further refined to include age, education level, annual income, work status, health insurance, and region. SES was established by considering annual income, education level, and health insurance status, respectively. Regarding health insurance status in Ireland, eligibility for entire public coverage through a medical card is based primarily on income, while health status and age are also considered. A medical card entitles the individual to primary care and hospital services free at the point of access; however, only 32% of the population are eligible for such coverage [24]. This eligibility structure causes inequalities for health services [25]. Thus, nearly 50% of Irish citizens seek out additional private, or voluntary, health insurance for quicker access to care [26]. Additional clinical information was obtained from the survey components, including year of diagnosis and cancer stage. Further information on the overall study design, participant recruitment, and sampling strata can be found in Supplementary 1.

### Data collection

Semi-structured qualitative interviews were conducted between January and March 2023 via Microsoft Teams (General Data Protection Regulation compliant) using a topic guide developed from the analysis of the baseline qualitative study [23] and baseline and follow-up survey study [22] by two qualitatively trained researchers (CM, CC). The topic guide included questions to explore women’s experiences and perspectives of BC care since the easing of COVID-19 government restrictions, including unmet needs, patient priorities, and recommendations specific to BC care. The topic guide was adjusted by removing, adding, or rewording questions during the interview process, a process known as reflexivity in qualitative research, which reduces researcher bias in data collection [27]. The final topic guide can be found in Supplementary 2. The interviews were anonymised and transcribed verbatim.

### Data analysis

Interviews were analyzed using a codebook approach to thematic analysis within NVivo software, which identifies themes early in the analysis process and subsequently maps concepts around central patterns or relationships within the data [28]. The codebook approach utilizes both deductive reasoning (e.g., the creation of the topic guide as a preliminary codebook, aligning with the research objectives) and inductive reasoning (e.g., the addition of topics and codes as the interviews was conducted). The codebook approach to thematic analysis was conducted using the following steps: identifying existing code sources/code development; familiarization with new data, generating additional codes, identifying patterns around codes for themes, reviewing themes, defining and naming themes, and producing the report [28, 29]. The data was organized by themes, summarized by sub-themes, and illustrated with quotations. Furthermore, cross-tabulation within NVivo was used to explore any variation in themes and sub-themes by SDH to associate patterns in variation. Evident variations were identified with a difference in proportion greater than 20%. SDH were then interpreted within the corresponding themes and sub-themes.

### Ethical approval

Ethical approval was obtained from the Office for National Research Ethics Committee in Ireland (20-NREC-COV-078). Participation was voluntary, and participants were able to withdraw their consent at any point throughout the research study.

## Results

### Participant characteristics

The follow-up interview study included 28 of the original participants which was a 76% response rate from the baseline interview study. There were nine women who were either lost to follow-up, uninterested, or deceased. Table 1 presents the clinical and demographic data of participants interviewed at follow-up. The average age for women in the study was 61.7 years (standard deviation (SD) = 12.3). Over half of women were diagnosed prior to 2020 (57%) and the majority of women reported an early stage (e.g. stage I–II) diagnosis (68%). Fifty-four percent of women were highly educated, and 68% of women reported not working due to unemployment, retirement, or as a result of BC. Half of women (50%) reported an annual household income of less than €40,000. Furthermore, 39% (*n* = 11) of women were categorized as high SES, while 61% (*n* = 17) of women were categorized as low SES. Supplementary 3 presents the refined SDH sampling strata by participant.

**Table 1:** Participant demographics (SDH) of the women with BC interviewed for the study (*N* = 28)

	*N* (%)
Year of diagnosis	
2020	10 (36%)
2018–2019	6 (21%)
2015–2017	12 (43%)
Cancer stage at diagnosis	
Stage 1	8 (29%)
Stage 2	11 (39%)
Stage 3	5 (18%)
Stage 4	2 (7%)
N/A	2 (7%)
Education	
Primary	4 (14%)
Junior/intermediate certificate	3 (11%)
Leaving certificate	5 (18%)
Diploma/certificate	6 (21%)
Third level/post-graduate degree	9 (32%)
Other	1 (4%)
Annual household income	
Below 20 K	4 (14%)
20-40 K	10 (36%)
40-60 K	3 (11%)
60-80 K	1 (4%)
80-100 K	1 (4%)
Above 100 K	2 (7%)
N/A	7 (25%)
Work status	
Employed	9 (32%)
Unemployed/ homemaker/ retired	12 (43%)
No longer working as a result of breast cancer	6 (21%)
Other	1 (4%)
Insurance status	
Private health insurance	11 (39%)
Medical card	9 (32%)
Both	6 (21%)
None	2 (7%)
Region	
In Dublin city/ county	11 (39%)
In a city other than Dublin	2 (7%)
In a town (1500 +)	5 (18%)
In a village	2 (7%)
In open countryside	8 (29%)

### Themes

The three main themes, along with their sub-themes and corresponding codes, are described narratively below, and also summarized in Table 2. SES was the only SDH that was associated with varied themes and sub-themes; therefore, SES is integrated narratively when evident.

**Table 2:** Themes, sub-themes, and codes derived from using a codebook approach to thematic analysis

Themes	Sub-themes	Codes
1. Unmet needs in BC care (*n* = 26)	Routine care fall-out* (*n* = 21)	Delays/cancellations Lack of contact with BC care team Medication difficulties Breast reconstruction
	Mental health (*n* = 18) Physical well-being (*n* = 13)	Anxiety, depression Fear of cancer recurrence Co-morbidities Fatigue Pain
	Financial support* (*n* = 10)	Loss of income Transportation Inability to pay for healthcare
2. Patient priorities for BC care (*n* = 28)	Cohesion* (*n* = 28)	Multiple clinic locations Continuity across multidisciplinary care Efficiency in appointments
	Communication* (*n* = 28)	Consistency and understandability Fall-out from routine care Telemedicine Designated healthcare contact
	Empowerment within BC care* (*n* = 20)	Personalisation Education and knowledge Self-management BC pathway
3. Recommendations for BC care (*n* = 28)	Transition from active treatment (*n* = 17)	Continued contact with BC care BC pathway awareness Cancer support centres
	Support resources (*n* = 18)	Financial aid Transportation Bras and wigs
	Telemedicine (*n* = 7)	Improve communication Transportation barriers Costs

^*^Difference in experience by SES

## *Unmet needs* in BC health care

Most women (*n* = 26, 98%) mentioned at least one on-going unmet need specific to their BC health care, which negatively impacts their overall well-being since the onset of the COVID-19 pandemic.

### Unmet needs in routine care for BC

Post-treatment care (i.e., routine care) includes follow-up appointments, exams, scans, and other tests that occur after active treatment, and most women reported an unmet need regarding their routine care (*n* = 21). Many women experienced persisting delays and/or cancellations (*n* = 16) since the pandemic. A higher proportion of women with low SES reported disruptions (*n* = 12, 71%) compared to women with high SES (*n* = 4, 36%) for routine appointments. Overall, these reported delays and/or cancellations were typically rescheduled and/or modified; however, women were dissatisfied without in-person annual mammograms: “Now, it was a drawback not being able to have my mammogram for two years and, well, I was keeping up with, you know, with my examinations myself, so I was kind of half OK, you know, that if anything was there, I would have felt it.” (P6, low SES).

Regarding routine care, many women expressed difficulty contacting their BC team (*n* = 17). Additional concerns included delays with breast reconstruction procedures (*n* = 6) and BC medication (*n* = 11). A higher proportion of women from low SES backgrounds reported medication difficulties (*n* = 9, 53%) compared to women from higher SES background (*n* = 2, 18%), including disruptions with supply of BC medications and a lack of support for the side effects and consequences from tamoxifen: “When I was given tamoxifen, I wasn’t aware that other medications could actually reduce its’ effectiveness. That was something I discovered during the year, myself, on my own. So I had to bring that to the attention of my doctors and request that they be changed.” (P8, low SES).

### Unmet needs in mental and physical healthcare

The combination of experiencing COVID-19 and having BC exacerbated both physical health and mental health for women, regardless of SES: “But the last six or eight months, again, I have felt very un-well and it just can’t be gotten to the bottom of, medically, you know… I don’t know whether it’s right or wrong or appropriate to attribute it to COVID or post-pandemic or is it just a fact of life post cancer?” (P28, high SES).

For physical health (*n* = 13), women noted developing pain, co-morbidities, and fatigue which was attributed to the disruption and lack of access to healthcare services during the pandemic: “That side of my arm and breast is very sore, so maybe if I was going to physio, it mightn’t be so bad. It’s hard to lift up that arm. It’s very, very sore.” (P16, low SES).

For mental health (*n* = 18), women expressed persisting depression, anxiety, and fear of cancer recurrence, which they believed would have improved post-pandemic, if they were able to access treatment: “Now I find in the last sort of six months, I’m struggling mentally. I’m a bit overwhelmed at times. I think that would be the best way to put it. And you know, the impact now of it, I suppose, is really taking effect now because you are dealing with the treatments and stuff like that. (P2, low SES).

However, these mental health needs were infrequently addressed through their BC care: “But they need to be more aware of the mental… health side of it as well. I don’t think there’s anything, well look, there is nothing done, there’s nothing there for it. That’s not part of the treatment plan and I think it should be.” (P7, low SES).

### Unmet financial support to access BC healthcare

Throughout the pandemic, some women experienced financial difficulties (*n* = 10), including disruption to work and income due to BC and/or COVID-19, resulting in an inability to pay for BC services and medications, and transportation issues. A higher proportion of women with low SES reported financial difficulties (*n* = 9, 53%) compared to women with high SES (*n* = 1, 9%): “And I wasn’t working, so I had no money at all. And so, I got sorted out. Well, it took a while and I think that’s hard on people because I had to like, actually beg for a medical card because I wasn’t even going to get the operation because I had no money to pay for an operation.” (P1, low SES).

The lack of financial support is still evident and on-going post-pandemic: “I have the hormone medication, the breast cancer one. So it is, that’s a bit of a pain every three months, having to pay that.” (P13, low SES).

## Patient priorities for BC healthcare

Women proposed priorities for their BC care (*n* = 28); which were personalized and included improving their health-related anxiety and overall well-being.

### Cohesion in BC healthcare

Cohesion among multidisciplinary BC care (e.g., oncology, surgery, radiotherapy, GP, physiotherapy, psychology, and pharmacy), spanning from diagnosis through post-treatment care, was a top priority for all women (*n* = 28). However, women’s experience with cohesive BC care varied; the majority of women described adequate BC cohesion (*n* = 18); however, of the women who described poor BC cohesion (*n* = 10), a higher proportion was of low SES (*n* = 8, 80%): “It didn’t feel linked… it almost felt a bit like a conveyor belt system. And you know…you could be with radiology and you might say something or have a concern and they’d be like, well, that’s not really our department. So you need to phone oncology.” (P7, low SES).

Women with poor BC cohesion expressed that the different elements for BC care (e.g. radiology, oncology) were not linked and those who attended appointments in varying BC clinics addressed concern regarding clarity in patient information: “When you’re coming in as a patient, they should know your history… Like I’ve been asked, ‘So you had cancer on the left?’ And then I said, ‘Yeah but I had double mastectomy.’ ‘Oh, I didn’t know that’, you know? They’re the type of things you should know as a doctor or a nurse…you should read the notes. They should have a preliminary page that says this patient has had this, this, and this.” (P3, low SES).

On the other hand, women expressed better cohesion with continuity and on-going monitoring with BC health professionals post-treatment: “Now I’m really lucky because we know they’re there. So, for instance, next month I’m having… a breast CT…so I’m kind of back into six-monthly checks now again. So that’s where I am at the moment, [I’m] being watched quite carefully now.” (P23, high SES).

Many women discussed the importance of efficiency with appointments (*n* = 13), including timely results and less delays: “I think the fact that the hospital…quite quickly geared up to being very, very efficient and they dealt with people when they arrived and soon as you were kind of finished, you were let go. There wasn’t any of the delays that would have been before.” (P24, high SES).

### Communication with and between BC healthcare providers

Proper communication, including consistency and understandability, with health professionals was another top priority for women interviewed in the study (*n* = 28). All women who expressed adequate cohesion in their BC care attributed it to good communication (*n* = 18): “Since [the pandemic], it has been the same, consistent. If they give me an appointment, it goes ahead and there’s no changes. If you ring them… they answer the phone and you get on to them and you know the details are there and so everything is fine from that point of view.” (P21, high SES).

However, poor communication was a common concern for some women (*n* = 10), and women’s experience with poor communication was attributed to the unmet need of fall-out from routine care: “It’s communication. It always comes back to communication, doesn’t it? And if you had somebody that you could just have a 5-min conversation with an’ it’s kind of like, just give me your opinion. Hear me. Hear me. First of all.” (P18, high SES).

To enhance communication, many women mentioned the use of telemedicine (*n* = 21), especially during the pandemic when in-person appointments were limited: “To know that there was somebody picking up your file, looking at it, picking up the phone and checking in with you. It was very reassuring.” (P6, low SES).

Indeed, there were differences in women’s experiences with communication. Similar to cohesion, 80% (*n* = 8) of women who expressed poor communication were of low SES, even with the use to telemedicine: “The phone call, like I said, you were just talking… there were no personal details. You know, you couldn’t show [them anything] over the phone.” (P9, low SES).

Additionally, women described the role of a BC nurse, junior doctor, or GP as an integral component to their BC care to enhance both communication and cohesion: “That continuity of care was very important… I found my oncology link nurse was extremely supportive… And I got that. But I imagine not everyone probably is that lucky.” (P20, high SES).

### Patient empowerment in BC healthcare

Empowerment was another top priority, and most women (*n* = 20) described the ability to be an active participant in their BC care. The personalization of BC care enhanced patient empowerment: “Everybody has different needs and different wants. And what I would find satisfactory, somebody else wouldn’t, you know?” (P10, low SES).

Furthermore, proper education and knowledge on BC treatment and results also improved mental well-being: “They really kind of involved me in a sense, showed me the evidence. And that really made a difference. I mean, that melted away any lingering anxiety I had. Now I’m just a new person. That could make a huge difference to somebody.” (P8, low SES).

However, several women did not experience empowerment and involvement within their BC care (*n* = 5), which was more common among women of low SES (*n* = 4, 80%). This lack of control caused health-related anxiety and worry: “It’s the effects of all the other things, you know? I find that a bit problematic… It’s a struggle. I don’t know what anyone even could do about it because I don’t know even myself.” (P1, low SES).

Women also discussed the importance of managing their BC care by understanding their specific pathway for treatment and care, and being aware of next steps: “I had my plan set out for me from the beginning of where we were going. Chemo, surgery, radiation. So you knew all that, which was great. You know, you weren’t second guessing it all the time. You knew you had a plan and the plan was going to plan.” (P2, low SES).

## Recommendations to improve BC healthcare

Considering their unmet needs and priorities for proper and personalized BC care, all women (*n* = 28) proposed improvements and recommendations to enhance BC care moving forward post-pandemic. Most of the recommendations addressed an unmet need and/or a patient priority.

### Transition from active treatment to post-treatment

To address the unmet need of routine care fall-out, many women (*n* = 17) suggested ways to improve the transition from active to post-treatment, a period of time when women feel abandoned from their habitual BC care. Women proposed continuity in care through continued contact with a designated BC nurse: “I think that could help a lot of people out, if you could ring the nurse and they could tell you what’s going on. It might be something very simple or, you know…it’s something that’s part of [the BC] because, as you know, the side effects are massive from medications.” (P17, low SES).

Women expressed the desire for clear communication on their BC care plan post-treatment: “I didn’t feel that they gave you…a little pamphlet or booklet or something that could give you directions if you have anything, anxieties… How do you get back into your normal life? And how do you deal with maybe upcoming events, something like that? It was just like dead stop.” (P2, low SES).

The promotion of local cancer support centers from the BC care team was a common suggestion to enhance the transition from active treatment to post-treatment: “There should be something, a follow-up from your treatment, as in a nurse even saying to you, ‘look, I think you should contact your local [support centre]’ or something but like I, I literally finished my treatment and that was it.” (P4, low SES).

Likewise, the use of local cancer support centers offered women supportive care to address physical and mental health unmet needs: “That psych-oncologist was brilliant and she arranged… she gave me the name and number of somebody in [support centre] to ring, which I did. And she said, ‘I think these services, these things, the thrive and survive… this would be really beneficial for you.’” (P18, high SES).

### Support resources for BC care

In addition to support centers, many women (*n* = 18) expressed the importance of support resources for their BC care to improve the transition from active treatment. To address the unmet need of financial support within BC care, women (*n* = 9) suggested ways to improve the barrier of healthcare costs specific to BC: “Look, there’s definitely…grants and stuff like that. I never chase them because they made it too difficult for you to access. When you’re in the midst of a diagnosis and you’re trying to process everything, the last thing you want to do is go through your emails, try find pay slips and try to find, you know, bank statements…a letter from your oncologist should be enough. You know?” (P7, low SES).

Women also recommended ways to improve the patient experience with general issues such as transportation (*n* = 8): “And I do think the volunteer drivers with the [support centre] that’s a massive plus. That’s how I used to get in because I would be very dopey when I finished treatment, so I wasn’t, it wasn’t safe for me to drive, so they were huge resource … and I wasn’t made aware of that in the hospital.” (P19, high SES).

Specific to BC, women discussed BC specific resources such as bras and wigs (*n* = 9); however, there was limited knowledge on the accessibility and availability of such resources: “I’m only talking about what’s available locally… I don’t think there are those things here. Even down to…getting a proper fitting bra or where to go for it… I didn’t seem to realize that the mastectomy bra… you get those cheap or free for your first one.” (P27, high SES).

### Telemedicine

The use of telemedicine was common throughout the pandemic (*n* = 21), and women recommended the adaptation of telemedicine moving forward post-pandemic when feasible (*n* = 7): “The use of technology has been…a positive. If I was to turn around and say, ‘can I have a video, you know, a phone consultation?’ I think most of the time, it wouldn’t be a problem if you were to ask for that rather than just having an actual face-to-face.” (P23, high SES).

More so, the adaptation of telemedicine can eliminate transportation barriers and reduce time spent waiting for appointments: “They were useful in the pandemic in that you couldn’t physically be in the same spot. You know, we were in lockdown. And I actually think sometimes it’s better to be able to do that rather than going up to spend 3 or 4 h in the hospital waiting to speak to somebody.” (P3, low SES).

## Discussion

The current study found that women with a diagnosis of BC are experiencing many unmet needs associated with their BC care post-pandemic. Unmet needs included disruption and discontinuation of routine BC care, a lack of treatments and support services to address women’s mental and physical well-being, and a lack of financial support for those women of low SES to help them access and obtain BC care. Considering such unmet needs, women identified their priorities for receiving adequate BC care and further proposed recommendations for improving BC care in the future. Cohesion within BC health care delivery and improved communication among BC healthcare providers were considered top priorities, both of which were perceived to empower women in managing their BC care. The following three recommendations addressed unmet needs and patient priorities: (1) improving the transition from active to post-treatment care, (2) enhancing and promoting support resources, and (3) appropriate adaptation of telemedicine.

Unmet supportive care needs were common for all BC patients throughout the pandemic, including physical and psychological needs, communication with clinicians, health system information needs, and other financial and social needs [30]. A previous quantitative study conducted during the pandemic found that unmet needs for BC survivors can be addressed with either comprehensive care or psychological and emotional support and women who reported more unmet needs also reported a significantly lower quality of life [15]. The results of our study found that women of low SES experienced greater disruption to routine care and increased financial difficulties specific to BC, which is consistent with research conducted prior to the pandemic [31]. It is likely the pandemic exacerbated pre-existing socio-economic inequalities in BC care; therefore, women who experienced greater unmet needs should be reintegrated into routine BC care along the entire cancer continuum [22].

The identification of patient priorities for personalized BC care ensures equality in BC care [32]. The women in our study identified cohesion and communication as top priorities; however, poorer cohesion and communication were both common for women from lower SES backgrounds. Personalized BC care should address comprehensive continuity for all women, regardless of SES, to improve equity in healthcare services. Women in this study proposed improving the transition from active to post-treatment by having a designated, or liaison, healthcare professional to support them with the transition. Research has shown the multidisciplinary benefits of a liaison nurse for cancer care, including physical, psychosocial, and communicative outcomes [33]. Cancer support centers also improve the transition from active treatment by providing cancer survivors a social and community network to address multidisciplinary needs [34]. However, the pandemic created barriers towards accessing such resources. Women should be made more aware of the availability of these centers, and other supportive care, directly from their BC care team.

Providing financial aid and transportation means to women in need, especially women from low SES backgrounds, can address health inequalities specific to accessing and obtaining BC care [35]. Strategies from a health systems level for reducing cancer-related inequalities include enhancing patient navigation along the cancer continuum and integrating telemedicine for routine care [36]. Furthermore, transportation barriers and auxiliary costs can be addressed with telemedicine, which was a widely utilized practice during the COVID-19 pandemic [37]. In addition, telemedicine can improve communication with continued contact with BC health professionals. As BC services recover from the COVID-19 pandemic, consideration should be given to the use of telemedicine in BC care and how it could be used more effectively to support women.

## Strengths and limitations

The study has a number of strengths including the large number of women interviewed and the selection via stratified purposive sampling to ensure diverse representation. This study is one of few studies to associate SDH, in particular SES, with BC care experience. The interviews were conducted immediately following COVID-19 government restrictions; therefore, they were timely and represent experiences of the transition from pandemic restrictions. There is limited research post-pandemic from the patient perspective; therefore, it addresses an evidence gap. However, there are several limitations to the study regarding generalizability. The participants do not represent all women living with a diagnosis of BC. The study was conducted in Ireland, a country which experienced severe and longer periods of restrictions compared with other countries [38]. Ireland remains the only country within the European Union without universal healthcare, and health inequalities have been associated with health insurance status and SES [39]. To enhance health equality in BC care, the findings from this research, in tandem with previous related research conducted during the pandemic [22], suggest that all women with a diagnosis of BC should be entitled to a medical card to assist with healthcare costs, if needed. Despite differences in health care systems, women with BC may be experiencing similar unmet needs across different countries and further research across countries with varying health care systems is needed to fully understand unmet needs for women with BC post-pandemic and inequities in these unmet needs. Additional future research may include comparing individual SDH characteristics to determine what SDH characteristics have a greater influence on women’s experience with BC care.

## Conclusion

The pandemic has impacted BC services considerably for women in Ireland with BC, and this study has identified a range of unmet needs in BC care, patient-centered priorities, and recommendations for addressing these unmet needs. These priorities and recommendations align with the goals of the national cancer strategy, which aims to put structures in place to allow for increased patient involvement in the delivery of BC care going forward [40]. As health systems are recovering from the COVID-19 pandemic, the emphasis should be on both restoring access to BC care and improving the quality of BC care to achieve the best possible health outcomes for women living with and beyond a diagnosis of BC. Particular consideration needs to be given to those women from lower socioeconomic groups, in order to reduce health inequalities, which have been further exacerbated by the pandemic. As health systems are recovering from the COVID-19 pandemic, an emphasis to restore and enhance better BC care should be essential, with consideration and emphasis from the patient perspective.

### Supplementary information

Below is the link to the electronic supplementary material.

Supplementary file1 (DOCX 26 KB)
