# Supplementary material for: Lessons learned from COVID-19: improving breast cancer care post-pandemic from the patient perspective
Source: Support Care Cancer. 2024 May 10;32(6):338. doi: 10.1007/s00520-024-08540-0 (PMC11087304; doi:10.1007/s00520-024-08540-0)
Supplement: Supplementary file 1 — Supplementary file1 (DOCX 26 KB) [file 520_2024_8540_MOESM1_ESM.docx]

Supplementary 1. Overall study design, participant recruitment, and sampling strata

The overall research study utilises a prospective explanatory sequential design,[37] including two timepoints. The first timepoint (baseline) corresponds with COVID-19 infection and government restrictions in Ireland. Initially, surveys were distributed starting in September 2020 and completed in April 2021, followed by interviews which were conducted from April 2021- May 2021. The second timepoint (follow-up) corresponds to the easing of government restrictions; surveys were distributed to participants again starting in June 2022 and completed in August 2022. Follow-up interviews were conducted early 2023.

Women were eligible to participate in the cohort study if they had a diagnosis of BC within the past 5 years, were living in Ireland, were over 18 years of age, were English speaking, and had no known serious psychiatric conditions. Women were recruited through a social media campaign, and surveys were also distributed through BC centres via nonprobability sampling techniques. Informed consent was obtained by all participants included in the study, and data were self-reported.

For interview recruitment at the baseline timepoint, women from the survey study (N=387) were categorized into strata by SDH including age, region, health insurance status, and education, and also by clinical characteristics including time since cancer diagnosis. Women were purposively sampled within these strata to ascertain any variation in experiences of COVID-19; 63 women were invited to take part in the initial interviews and 37 women participated. The same women were invited to participate in the follow-up interviews.

Additional data on SDH were extracted from the follow-up questionnaire (N=272)

and socio-demographic characteristics were further refined from the baseline interview to ensure timeliness and accuracy. Socio-demographics included: age; education; income; employment; health insurance status; region; and socio-economic status (SES). For age, participants were categorised as under 65 vs. 65 and older. For education level, participants were categorised as low (primary/secondary) education vs. high (diploma/third-level/postgraduate) education. For income, participants were categorized as low (below 40,000 euro per annum) income vs. high (above 40,000 euro per annum) income. For employment status, participants were categorized as employed vs. non-employed (e.g. unable to work sickness/disability/ student/ unemployed/ retired). For health insurance status, participants were categorised as with or without private insurance. For region, participants were categorised as urban (living in a city/ town) vs. rural (living in a village/ countryside). For SES, participants were categorised as either high SES or low SES. SES was established by amalgamating income level, level of education, and health insurance status, respectively in the following order. Women were categorized as low SES primarily by low income, however low level of education and no private health insurance was addressed as well; women were categorized as high SES primarily with high income, however high level of education and private health insurance were addressed as well.

Supplementary 2

Topic guide

| **Interview themes** | **Pre-codes** |
| --- | --- |
| **1. Reflection** | - Lasting impact of COVID-19 - Daily life - QoL/ psychosocial well-being |
| **2. Breast cancer experience** | - Current status - Access to appointments/ treatments/ medications - Access to healthcare professionals - Modes of care delivery - Multidisciplinary care / support services - Health concerns |
| **3. Patient-centered priorities** | - Difficulties /negatives / barriers - Benefits/ positives/ facilitators - Recommendations/ solutions |

**1. Reflection from the COVID-19 pandemic**

Reflecting back on the last couple of years, since the onset of COVID-19, how do you feel the pandemic has affected your life?

Probes:

- Daily life, long-term impacts, work life, financial security, psychosocial well-being, quality of life, etc.

How do you feel having breast cancer affected your experience throughout the pandemic?

**2. Breast cancer experience within the last year**

- - Current status
    - When we last spoke, you were [STATUS]. Can you tell me where you are now on your breast cancer journey?
      - If applicable- how do you feel about any changes that may have occurred in your prognosis over the last several years?
    - What is next for your cancer care?
    - Regarding breast cancer, what have been the main impacts of the COVID-19 pandemic? Long-term impacts?
  - Access to appointments/ treatments / medications
    - Can you tell me about your appointments/ treatments in the past year, since the restrictions have been lifted? How were these different compared to your appointments/ treatments during the lockdown phases of COVID? Access to medications?
    - If you experienced major delays and/or cancellations during the pandemic, have these appointments been rescheduled and carried out? Please elaborate.
  - Access to healthcare
    - How has your interaction and communication with your breast cancer care team changed within the last year, since the restrictions have been lifted?
    - Do you currently have a designated individual to contact with any questions or concerns you may have regarding breast cancer, someone who you feel would respond promptly?
      - If yes, how does this contact influence your BC care?
      - If no, do you think having a designated individual to contact with questions or concerns would benefit you? Please elaborate.
  - Patient experience:
    - - Could you tell me about your different cancer care elements and if and how they may be linked?
      - Can you tell me about how you to manage your cancer care? How can this be improved?
      - Do you feel empowered in your cancer care? How confidence are you in managing your BC care?
      - What role does your GP play into your breast cancer care?
  - Modes of care delivery
    - In what setting do you receive your current breast cancer care? In-person, online? How do you feel about this delivery of care?
  - Multidisciplinary care / support services
    - What support services (e.g. psychologists, physical therapy) are available/ would you like to be available to you through you breast cancer clinic? Has this changed in the last year, since the lifting of COVID-19 restrictions?
    - (if applicabe): How can the transition out of active treatment be improved for you?
  - Health concerns
    - Can you tell me about any concerns or worries you may have about breast cancer care moving forward?

**3. Patient-centered priorities**

- - Difficulties /negatives / barriers
    - What has been most difficult for you during the pandemic? Breast cancer specific?
    - What were the major negatives/barriers to your breast cancer care since the COVID-19 pandemic?
  - Benefits/ positives/ facilitators
    - Can you tell me about any positives in your life throughout the pandemic? Breast cancer specific?
    - What were the major positives/facilitators to your breast cancer care since the COVID-19 pandemic?
  - Recommendations/ solutions
    - Given your experience of living through a pandemic, what do you think could have been done to support you better?
      - Different for women with BC vs other patients
      - Financial support?
    - What are your top priorities for receiving adequate and personalised breast cancer care? What do you value most in your breast cancer care?
    - Based on your experience of being treated for breast cancer during the COVID-19 pandemic, what would you recommend…
      - - To other women who have breast cancer?
        - To their caregivers?
        - To the healthcare professionals?
        - To policymakers who make decisions about the organization of healthcare in Ireland?
        - To patient- or community-based organizations?
        - Anyone else?

**Conclusion**

Is there anything else you would like to add regarding our discussion today?

Supplementary 3

Social determinants of health (SDH) strata used for analysis within themes

| ID | Socio-economic status | Age (years) | Insurance status | Region | Education | Income | Work status |
| --- | --- | --- | --- | --- | --- | --- | --- |
| P1 | Low | < 65 | No private | Urban | Low | Low | Non-employed |
| P2 | Low | < 65 | No private | Urban | High | Low | Employed |
| P3 | Low | < 65 | Private | Urban | Low | - | Non-employed |
| P4 | Low | < 65 | No private | Rural | Low | Low | Non-employed |
| P5 | Low | < 65 | Private | Rural | Low | Low | Non-employed |
| P6 | Low | < 65 | No private | Rural | High | Low | Employed |
| P7 | Low | < 65 | Private | Urban | Low | - | Non-employed |
| P8 | Low | < 65 | Private | Rural | High | Low | Non-employed |
| P9 | Low | ≥ 65 | No private | Urban | Low | Low | Non-employed |
| P10 | Low | ≥ 65 | No private | Urban | Low | Low | Non-employed |
| P11 | Low | ≥ 65 | Private | Urban | Low | Low | Employed |
| P12 | Low | ≥ 65 | No private | Urban | Low | - | Non-employed |
| P13 | Low | ≥ 65 | No private | Rural | High | Low | Non-employed |
| P14 | Low | ≥ 65 | Private | Urban | Low | Low | Non-employed |
| P15 | Low | ≥ 65 | No private | Urban | High | Low | Non-employed |
| P16 | Low | ≥ 65 | No private | Urban | Low | - | Non-employed |
| P17 | Low | < 65 | Private | Urban | High | Low | Employed |
| P18 | High | < 65 | Private | Urban | High | High | Non-employed |
| P19 | High | < 65 | Private | Urban | High | High | Employed |
| P20 | High | < 65 | Private | Urban | High | High | Employed |
| P21 | High | < 65 | No private | Rural | Low | High | Employed |
| P22 | High | < 65 | Private | Rural | Low | High | Employed |
| P23 | High | < 65 | Private | Rural | High | High | Non-employed |
| P24 | High | ≥ 65 | Private | Urban | High | High | Non-employed |
| P25 | High | ≥ 65 | Private | Urban | High | - | Employed |
| P26 | High | ≥ 65 | Private | Urban | High | - | Non-employed |
| P27 | High | ≥ 65 | Private | Rural | High | Low | Non-employed |
| P28 | High | ≥ 65 | Private | Rural | High | - | Non-employed |
